# Perceived barriers to psychiatric help-seeking in South Korea by age groups: text mining analyses of social media big data

**DOI:** 10.1186/s12888-022-03969-1

**Published:** 2022-05-13

**Authors:** Hwo Yeon Seo, Gil Young Song, Jee Won Ku, Hye Yoon Park, Woojae Myung, Hee Jung Kim, Chang Hyeon Baek, Nami Lee, Jee Hoon Sohn, Hee Jeong Yoo, Jee Eun Park

**Affiliations:** 1 Division of Public Health and Medical Service, Seoul National University Hospital, Seoul, Korea; 2 The Mining Company, VAIV, Seoul, Korea; 3 Department of Neuropsychiatry, Seoul National University Hospital, Seoul, Korea; 4 Department of Psychiatry, Seoul National University College of Medicine, Daehak-ro 103, Chongno-gu, Seoul, 03080 Korea; 5 Department of Neuropsychiatry, Seoul National University Bundang Hospital, Seongnam, Korea

**Keywords:** Mental health, Service, Barrier, Discrimination, Stigma, South Korea

## Abstract

**Background:**

The psychiatric treatment gap is substantial in Korea, implying barriers in seeking help.

**Objectives:**

This study aims to explore barriers of seeing psychiatrists, expressed on the internet by age groups.

**Methods:**

A corpus of data was garnered extensively from internet communities, blogs and social network services from 1 January 2016 to 31 July 2019. Among the texts collected, texts containing words linked to psychiatry were selected. Then the corpus was dismantled into words by using natural language processing. Words linked to barriers to seeking help were identified and classified. Then the words from web communities that we were able to identify the age groups were additionally organized by age groups.

**Results:**

97,730,360 articles were identified and 6,097,369 were included in the analysis. Words implying the barriers were selected and classified into four groups of structural discrimination, public prejudice, low accessibility, and adverse drug effects. Structural discrimination was the greatest barrier occupying 34%, followed by public prejudice (27.8%), adverse drug effects (18.6%), and cost/low accessibility (16.1%). In the analysis by age groups, structural discrimination caused teenagers (51%), job seekers (64%) and mothers with children (43%) the most concern. In contrast, the public prejudice (49%) was the greatest barriers in the senior group.

**Conclusions:**

Although structural discrimination may most contribute to barriers to visiting psychiatrists in Korea, variation by generations may exist. Along with the general attempt to tackle the discrimination, customized approach might be needed.

## Introduction

The global burden of mental illness is considerable. Recent research suggests that mental illness is on a par with cardiovascular disease in terms of the level of disability caused [1]. In the European Union, almost 50% of the general population reportedly develops a mental disorder during a lifetime, and the economic cost of mental illness is approximately US$2.5 trillion [2]. In 2017, the National Institute of Mental Health estimated that one in five adults live with a mental illness [3]. South Korea (hereafter, Korea) also has a high prevalence of mental illness. A nationwide epidemiologic survey of mental disorders in Korea showed that one-quarter of Koreans are affected by a mental disorder during their lifetime [4]. Furthermore, approximately 60% of respondents to another recent Korean survey self-reported having mental health problems during the previous year [5]. Because mental illness is often chronic and its prevalence is high, the associated economic burden may account for 4% of the overall gross domestic product in Korea, corresponding to 6.4 million dollars [6].

Although effective pharmacological and psychological interventions have been developed, globally, the proportion of people receiving treatment for a mental illness is low [7]. This discrepancy between the number of people who need a treatment and those who receive it is called the treatment gap, and in psychiatry this is a major problem. Several important barriers to seeking help for mental illness have been identified by previous research. One of these barriers is the stigma associated with mental illness. This is a combination of stereotyping, prejudice, and discrimination, manifesting as cognitive, affective, and behavioral responses toward people with mental illness [8]. A comprehensive review of quantitative and qualitative studies revealed that stigma has a negative effect on help-seeking, with concerns about disclosure being the most common stigma-related barrier [9]. Other barriers are structural, such as medical costs and accessibility, as well as poor mental health literacy [10]. Although structural barriers should be less important in Korea, due to universal coverage with a national insurance system, only 3.9% of all clinics are psychiatric clinics [11]. Mental health literacy is defined as “knowledge and beliefs about mental disorders which aid their recognition, management, or prevention” [12]. Better mental health literacy results in more people having psychotherapy and increased adherence to psychiatric medication [13].

Although Korea is economically developed, the psychiatry treatment gap remains huge. An epidemiologic survey in Korea reported that only 22% of people with a mental illness seek professional help during their lifetime [4]. Despite the need for urgent action, previous studies on the barriers to psychiatric treatment in Korea have focused mainly on discrimination [14, 15] and stigma [16, 17]. Few studies have used quantitative methods.

Text mining (TM) is a novel technique that enables researchers to process an unprecedented amount of textual data by subdividing and extracting necessary information. Given the tremendous quantity of communications available on the internet, careful exploration of web-based information has the potential to provide valuable insight on specific issues. TM has proved to be a powerful tool in health research, particularly when combined with natural language processing (NLP) [18]. In psychiatry, where public discussion of mental illness is associated with stigma, TM and NLP have provided new opportunities for research [19]. For example, TM has been used to explore mental disorders, including depression [20], anxiety [21], substance abuse [22], eating disorders [23], and suicidal ideation [24]. Because TM can include both qualitative and quantitative study features, it has some advantages over conventional qualitative research and can access many different corpora.

Korea has an international reputation for its strong internet-access infrastructure [25]. Furthermore, according to a government report regarding internet usage in Korea, 91.7% of all Koreans and more than 99% of those aged 13–59 years old use internet services at least once a month [26]. This high accessibility and internet usage in Korea increases the value of research using TM methods.

In this study, we used text available on the internet (1) to look through characteristics of words relevant to psychiatry in comparison to internal medicine and surgery, (2) to investigate barriers to psychiatric treatment and (2) to evaluate the significance of these barriers in different age groups.

## Methods

### Data source

**Fig. 1:**
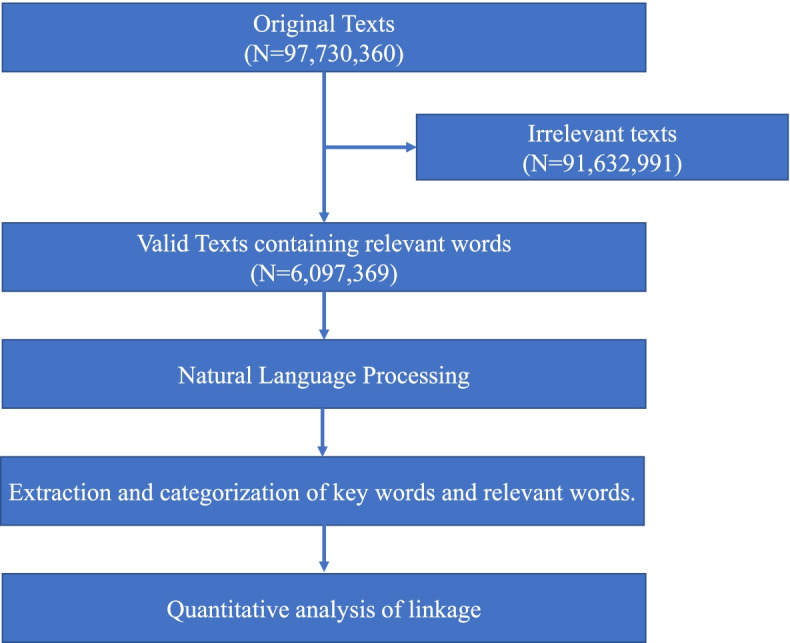
Flow chart showing the texts included in this study

Figure 1 shows the outline of the method. We obtained social media data from VAIV (Seoul, South Korea), one of the leading companies for social media analyses in South Korea. Data were collected from 1 January 2016 to 31 July 2019. Text was collected from web communities, social network services (SNS), and personal blogs because internet users commonly express their opinions using such sites. In Korea, web communities called ‘cafés’ are served by internet platform service providers such as Naver (http://www.naver.com) and Daum (http://www.daum.net). These cafés are easy to use, and anyone can create a new web community and invite people with the same interests to join. Personal blogs and SNS are also good sources of internet users’ ideas and opinions. Because data can be collected from Twitter quite easily, we included this SNS in our study. We excluded Instagram and Facebook, despite their popularity, due to the risk of disclosing personal information. We also included some text-based message boards that are not hosted by internet platforms.

### Data analyses

#### Natural language processing

Original text had to be analyzed automatically. Furthermore, many articles published online have errors in spelling and grammar. NLP allows computers to analyze language. The SOCIALmetrics™ engine, provided by VAIV, subdivided the original texts that were identified into sentences and morphemes after the external links and stop words were removed. Stop words include prefixes and suffixes. In a Korean sentence, prefixes and suffixes determine the meanings and grammatical functions of the words they are attached to.

For each word, the most appropriate combination of morphemes was tagged as parts of speech, such as noun or adjective. If expressions were difficult to analyze, they were paraphrased as simple words based on a normalized dictionary. Noun phrases were processed individually. For example, ‘big hospital’ was processed as a single item rather than ‘big’ and ‘hospital.’

After NLP, additional TM techniques were applied. Synonyms were transformed into representative words using the dictionaries installed in the analytical software. To simplify the data and the analysis procedure, noun groups and predicates were used as keywords and phrases, and levels of linkage among the extracted keywords were calculated. Keywords and phrases were categorized as shown in Table 1. The analysis procedure used in this study was developed from a dictionary containing 8,940 words, categorized into 9 major categories and 26 subcategories. For example, the keyword ‘schizophrenia’ falls into the major category ‘health’ and the minor category ‘illness.’

**Table 1:** Analyses of keywords

Major Category	Subcategory	Keywords
**Interest**		
	Lifestyle	Health, life, exercise…
	Crime	Gangnam station crime…
**Circumstance**		
	Season and weather	Spring, Winter…
	Time	Now, today, everyday…
	Place	Hospital, house, outside…
	Normal	Lecture, arrangement, birth…
	Special	Trauma, sexual abuse…
**Relation**		
	Age	Teens, twenties…
	Object	Alone, kids, friends…
	Relationship	Divorce, break-up…
	Life cycle	Adolescent, childhood…
**Consideration factor**		
	Personal	Service fee, side effect…
	Social	Record, discrimination…
**Health**		
	Physical	Skin, face, hair…
	Disease	Disease, early, cancer…
	Mental disorder	Stress, depression…
	Symptom	Headache, self-harm…
**Treatment**		
	Media	Program, internet…
	Institution	Health minister, prosecutor…
	General	Counseling, mental health…
**Predicate**		
	Positive	Good, helpful, grateful
	Negative	Bad, sick, difficult…
	Adjective	be, far, much…
	Verb	Receive, go, come…
**Non-predicate**		
	Noun	Person, task, idea…
**Group word**		
	Group word	Mental health

#### Corpus selection

Prior to focusing on psychiatry specifically, we identified words that were associated with ‘psychiatry,’ ‘internal medicine,’ and ‘surgery.’ To cover texts relevant to psychiatry as widely as possible, ‘mental health’ and ‘mental illness’ were settled as main words for their conceptual comprehensiveness. Then, words associated with the two words were selected based on the frequency. Texts containing selected words were included for further analyses. These words included ‘psychiatry,’ ‘mental health,’ ‘mental illness,’ ‘mental hospital,’ ‘psychological therapy,’ ‘distress,’ ‘treatment,’ ‘prescription,’ ‘depression,’ ‘suicide,’ and ‘antidepressant.’ We excluded specific disorders as they could bias the result. To enhance the specificity of the corpus, texts containing approximately 500 words that were associated with advertisements, such as ‘second-hand car,’ were excluded.

#### Categorization of barriers

Among the words associated with ‘psychiatry,’ we focused on those that described barriers to seeking psychiatric treatment. We categorized these words and read as many articles as possible to understand the contexts in which the words were used. The words were categorized at a structural and individual level by a TM professional, based on a theoretical multilevel conceptual framework suggested by Megan et al. [27]. At each level, the concept of stigma was subdivided into stereotype, prejudice, or discrimination subtypes. Because access to original texts was limited, it was impossible to subdivide them completely. However, the original framework was adapted to generate items that were sufficiently clear and reasonable.

#### Categorization of age groups

Although the writers’ demographic data could not be accessed, a part of corpus could be classified generally by age group, based on their web community. Some communities were considered to represent specific age groups with similar interests. Also, these web communities have cultural characters shared by the age group in question. For example, writers in the community for sharing information on university entrance exam were deemed teenagers. Texts from the web community ‘people searching for jobs seriously’ were likely written by 20- to 30-year-old job seekers as the government official employment was the main topic in the community. Texts from the web community ‘pregnancy and bringing up children café’ were likely written by 30- to 40-year-old mothers. Most members of café ‘elegant menopause’ may be 50-year-olds. In the long list of web communities, such communities that could be identified were selected for the further analysis. In addition, some message boards and internet portals classify their members according to age group. Therefore, these data were also analyzed separately for each age group.

## Results

A total of 97,730,360 articles were identified for the period in question. Table 2 shows the 20 keywords most frequently associated with ‘psychiatry,’ ‘internal medicine,’ and ‘surgery’ in this corpus. The word most frequently associated with ‘psychiatry’ was ‘information’ (7.8%; Table 2). The word most frequently associated with both ‘internal medicine’ and ‘surgery’ was ‘symptom.’

**Table 2:** The 20 words that were most frequently associated with psychiatry, internal medicine, and surgery

	**Psychiatry**		**Internal medicine**		**Surgery**	
No	**Keyword**	**Share**	**Keyword**	**Share**	**Keyword**	**Share**
1	**Information**	**7.80%**	Symptom	**13.40%**	Symptom	**12.60%**
2	Symptom	7.70%	Dysfunction	8.20%	Dysfunction	8.20%
3	Mind	7.40%	Medicine	6.90%	Condition	7.80%
4	Psychology	6.90%	Dysfunction	6.70%	Problem	6.60%
5	Idea	6.90%	Problem	6.40%	Effect	5.80%
6	Condition	6.80%	Condition	6.10%	Management	5.40%
7	Appliance	6.40%	**Information**	5.60%	Result	4.80%
8	Way	6.40%	Function	4.80%	**Information**	4.50%
9	Problem	5.80%	Food	4.60%	Drug	4.40%
10	Medicine	5.70%	Result	3.70%	Disorder	4.10%
11	Disorder	5.50%	Management	3.70%	Function	3.90%
12	Dysfunction	4.10%	Appliance	3.70%	Experience	3.90%
13	Behavior	3.50%	Usage	3.60%	Hobby	3.70%
14	Instance	3.10%	Effect	3.60%	Everyday life	3.70%
15	**First**	**3.00%**	Hobby	3.50%	Idea	3.70%
16	Anxiety	2.80%	Usual	3.40%	Usual	3.60%
17	Relationship	2.60%	Idea	3.10%	Mind	3.40%
18	Emotion	2.60%	Feeling	3.10%	Recent	3.30%
19	Result	2.60%	Mind	3.00%	Recovery	3.20%
20	Society	2.50%	Panic	2.90%	Personal	3.20%

Among the texts, 6,097,369 contained keywords associated with psychiatry, including 2,323,303 texts from web communities (36.4%), 1,896,239 texts from blogs (31.1%), and 1,963,827 texts from Twitter (32.2%).

We identified approximately 3,000 words associated with ‘psychiatry’ according to their frequency. Many of these words were linked to the topic of discrimination. A recurring theme was concern that disclosure of having a mental illness would be disadvantageous due to government policy or social stigma. Consequently, these words were classified in the ‘structural discrimination’ word group. These terms/words included ‘medical record,’ ‘public official employment,’ ‘buying insurance,’ ‘disadvantage,’ and ‘non-insurance’ (Table 3). Another recurring theme was stereotypes and prejudice associated with mental illness. This word group was labeled the ‘public prejudice group’ and included the terms/words ‘mad person,’ ‘negative attitude,’ ‘prejudice,’ ‘stigma,’ and ‘sympathy.’ Words expressing concern regarding adverse drug effects were classified in the ‘adverse drug effect’ word group and included the terms/words ‘adverse effect,’ ‘tolerance,’ ‘withdrawal symptom,’ ‘addiction,’ and ‘dependence.’ Words expressing concern regarding medical costs were classified in the ‘low accessibility’ word group and included the terms/words ‘medical fee,’ ‘medication fee,’ ‘expensive,’ ‘burdensome,’ and ‘counseling fee.’ Although these terms/words could have been classified in the ‘structural discrimination’ word group, we created the ‘low accessibility’ word group to highlight a distinct barrier to psychiatric treatment.

**Table 3:** The 10 words that were most frequently associated with each barrier

	**Structural discrimination**	**Freq**	**Public prejudice**	**Freq**	**Adverse drug effects**	**Freq**	**Low accessibility**	**Freq**
1	Medical record	14,690	Mad person	7710	Adverse effect	11,535	Medical fee	6361
2	Public official employment	4202	Negative attitude	6340	Tolerance	1841	Medication fee	1981
3	Buying insurance	2968	Prejudice	5587	Withdrawal symptom	1021	Expensive	1341
4	Disadvantage	2885	Stigma	935	Addiction	331	Burdensome	1149
5	Non-insurance	828	Sympathy	735	Dependence	176	Counseling fee	784
6	Disadvantage on job	699	Abnormal	549	Potent	158	Treatment cost	655
7	University	666	Finger pointing	337	Taking duration	97	Copay	394
8	Browse	465	Tag	245	Discontinuation	78	High cost	190
9	F code	435	Other’s view	226	Sequela	75	Subsidy	172
10	Entrance examination	218	Loser	184	Overdose	67	Poor	69

**Fig. 2:**
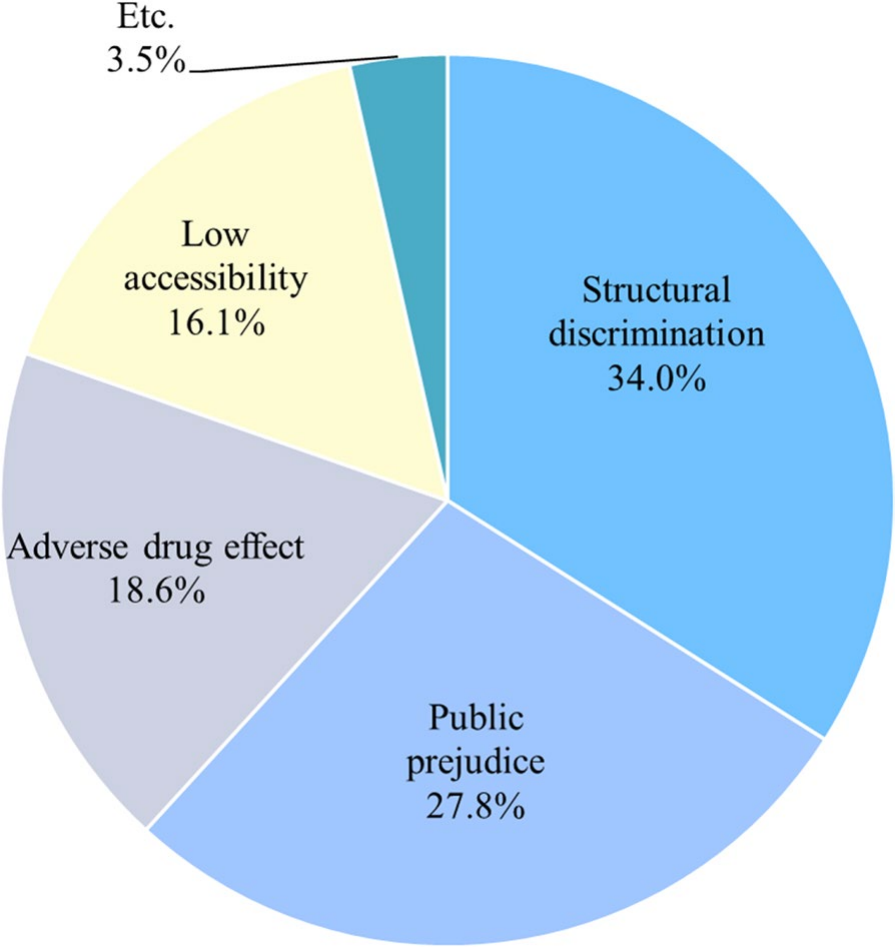
Barriers according to keywords

Figure 2 shows the frequency of keywords in each of the following categories: structural discrimination, public prejudice, adverse drug effects, and low accessibility. Structural discrimination was the greatest barrier to receiving psychiatric treatment, accounting for 34% of all of the keywords. Public prejudice was the next biggest barrier (27.8%), followed by adverse drug effects (18.6%), and low accessibility (16.1%). The 10 words that were most frequently associated with each barrier are shown in Table 3. In the structural discrimination section, ‘medical record’ was overwhelmingly the most frequently used term, followed by ‘public official employment,’ ‘insurance,’ ‘disadvantage,’ ‘non-insurance,’ ‘disadvantage in seeking jobs,’ ‘university,’ ‘browsing records,’ ‘F-code diagnosis,’ and ‘entrance examination.’ ‘Mad person’ was the most frequently used term in the public prejudice section, followed by ‘negative perception,’ ‘prejudice,’ ‘stigma,’ ‘sympathy,’ ‘abnormal,’ ‘finger-pointing,’ ‘tag,’ ‘other’s view,’ and ‘loser.’ Concerns about medication in the adverse drug effects section were clear from words/terms such as ‘adverse effect,’ followed by ‘tolerance,’ ‘withdrawal symptom,’ ‘addiction,’ ‘dependence,’ ‘potent,’ ‘taking duration,’ ‘discontinuation,’ ‘sequela,’ and ‘overdose.’ The low accessibility section included ‘medical fee’ followed by ‘medication price,’ ‘expensive,’ ‘burdensome,’ ‘counseling fee,’ ‘treatment cost,’ ‘copay,’ ‘high cost,’ ‘subsidy,’ and ‘poor.’

**Fig. 3:**
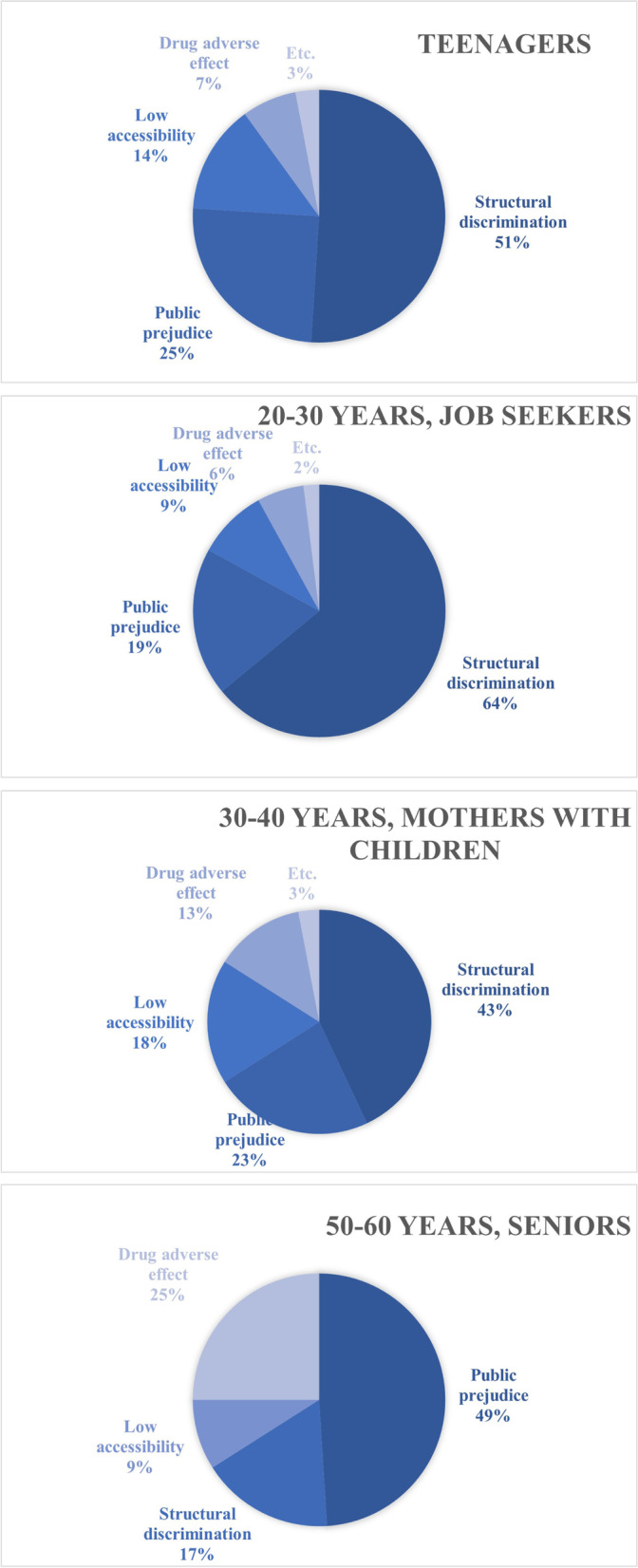
Barriers according to keywords, arranged by age group

Data from selected web communities were also separated into the following four groups: teenagers, 20- to 30-year-old job seekers, 30- to 40-year-old mothers with children, and 50- to 60-year-old seniors. Figure 3 shows the barriers to psychiatric treatment arranged by age group. Structural discrimination was the greatest barrier to treatment in the teenagers (51%), young job seekers (64%), and mothers with children (43%), followed by public prejudice, low accessibility, and adverse drug effects. However, in the seniors group, public prejudice (49%) was the greatest barrier to treatment, followed by adverse drug effects and structural discrimination. The seniors group was least concerned about low accessibility, whereas accessibility was of greater concern to the other groups than adverse drug effects. ‘Record’ was the word most frequently associated with barriers to treatment in all groups except the seniors group, whereas ‘university’ was only linked to the teenagers group, ‘public official’ was only linked to the jobseekers group, and ‘buying insurance’ was only linked to the mothers with children group. ‘Prejudice’ and ‘negative perception’ were only linked to the seniors group (Table 4).

**Table 4:** The five words that were most frequently associated with barriers, arranged by age group

Rank	Teenagers	Share (%)	20–30 years Job seekers	Share (%)	30–40 years Mothers with children	Share (%)	50–60 years Seniors	Share (%)
1	Record	25.9	Record	22.4	Record	22.1	Mad person	19.0
2	Mad person	14.4	Public official	15.6	Mad person	14.8	Adverse effect	16.7
3	University	10.6	Disadvantage	13.6	Adverse effect	10.9	Prejudice	13.1
4	Disadvantage	8.0	Mad person	7.1	Medical fee	9.3	Record	10.7
5	Medical fee	6.8	Medical fee	4.5	Buying insurance	5.1	Negative attitude	9.5

## Discussion

This study investigated the barriers to seeking help from psychiatrists in Korea. To the best of our knowledge, this is the first study to analyze these barriers using TM of internet big data within Korea. The results show that concerns about structural discrimination are the greatest barrier to seeking help, followed by public prejudice, adverse drug effects, and low accessibility. Interestingly, the greatest barrier is contrasting by age groups. While younger Koreans were more concerned about structural discrimination, older Koreans regarded public prejudice as the greatest barrier to seeking help.

Our results differ from those of previous studies. A study of barriers to mental health treatment based on the World Health Organization’s mental health surveys reported that attitudinal barriers to initiating and maintaining treatment are more important than structural barriers [28]. A study conducted in the United States reported similar results, that attitudinal factors were more important than structural factors in motivating help-seeking behavior [29]. In these studies, the structural barriers included limited insurance coverage, high medical fees, lack of information, lack of appropriate medical services, and problems accessing services (e.g., lack of transportation). The attitudinal barriers included personal beliefs such as mistrust of treatment, perceived stigma associated with having a mental disorder, and fear of involuntary admission to hospital. Some of the inconsistencies among these studies may be due to discrepancies in the research methods used and how the different barriers are understood.

Stigma is a controversial concept, and it is difficult to define [8]. The word ‘stigma’ may be used to convey different meanings, and the same meaning may be expressed using different words. To minimize the confusion associated with different interpretations of ‘stigma,’ studies have sought to standardize research using predefined concepts and tools to analyze data. Unlike the conventional top-down method, data mining features an inductive bottom-up approach. For this reason, studies must be compared using basic concepts. For example, the structural barriers identified by previous studies may include economic barriers and lack of accessibility (e.g., insurance coverage or travel to distant clinics), which were categorized as ‘low accessibility’ in our study. In previous studies, attitudinal factors may be equivalent to the conventional concept of ‘stigma.’ These factors were included under the headings ‘structural discrimination’ and ‘public prejudice’ in our study. Nonetheless, discrimination and negative attitude are distinct concepts, and in contrast to previous studies, we categorized them separately.

### Fear of discrimination

In Korea, medical costs are covered by the national insurance system. Diagnoses and treatment records are monitored to calculate the financial cost of this service. A major benefit of this system is that it increases accessibility by decreasing the cost of treatments. However, some patients worry that their medical records may be disclosed inappropriately. The Korean standard classification of diseases records mental and behavioral diagnoses in section ‘F’ and the so-called ‘F code’ is sometimes considered a ‘Scarlet Letter’ [30].

Corrigan et al. suggested that structural discrimination against people with mental illnesses may occur intentionally or unintentionally [31]. Opportunities to join some professions are restricted by law. For example, the law in Korea prohibits individuals with severe mental illnesses from becoming medical professionals, unless a psychiatrist approves their appointment. Similar restrictions apply to pharmacists, emergency medical technicians, and veterinarians. In addition, the employment of public officials may be terminated legally if a severe mental illness is diagnosed.

Discrimination against people applying for private insurance exemplifies unintentional discrimination because this may be justified commercially. Insurance companies frequently refuse to underwrite people with mental disorders. One reason for this may be that mental disorders are heterogeneous and difficult to diagnose precisely [32]. However, Kim [14] reported that people diagnosed with a mental disorder in Korea were unfairly rejected by insurance companies, regardless of their diagnosis. This clearly violates the spirit of the constitution of South Korea.

### Fear of stereotyping and prejudice

Public prejudice may include both internal stereotyping and prejudice against people with mental illnesses. Our results showed that younger people were more concerned about structural discrimination, whereas older people feared public prejudice. This reflects the different attitudes exhibited by each of these generations. Interestingly, some studies have reported a change in public attitudes toward mental illness. A meta-analysis of national surveys, conducted mainly in western countries, showed that attitudes toward mental illness have remained unchanged or worsened over the last few decades, despite considerable improvements in mental health literacy [33]. One study that investigated changing attitudes in Sweden over almost 40 years concluded that the stigma associated with mental illness had increased [34]. However, the authors of that study also reported that people who were younger than 20 years old had a more positive view of mental illness. Similar results were obtained from a recent national survey in Korea: respondents who were younger than 30 years old did not associate mental illness with stigma, although respondents who were older than 30 years of age did [4]. Our findings suggest that the different attitudes of each generation to mental illness reflects the particular interests of that age group rather than any stigma associated with mental illness. In developing interventions to tackle the barriers, different approaches to age groups may be necessary. For example, the young may need policies to guarantee the confidentiality in medical record. The seniors may need education on medication and its safety to improve help-seeking.

### Study limitations

This study had several limitations. First, although the TM method can scan a huge quantity of information, its results resemble an aerial photograph rather than a detailed map. Previously defined barriers to using mental health services could not be applied to our data. Therefore, it is difficult to compare our results with those from other studies. Second, barriers were categorized based only on words, and the same words were interpreted as having the same meaning, despite the possibility of different uses in different contexts. Third, although the internet is used widely in Korea, the population willing to communicate in cyberspace may differ from the general population, resulting in sampling bias. Fourth, although we have sought to cover the texts relevant to psychiatry as widely as possible, the selection process could be arbitrary to some extent. Although psychiatrically related, if the words were rarely mentioned they could be missed in the selection of words relevant to psychiatry. Finally, the analysis by age groups lacks quantitative robustness despite its insightful implication. As only a part of corpus was included, the representativeness of the selected could be limited. Also, communities were loosely classified into age groups based on their interest-group topics. Therefore, some texts in a community could be written by writers from other age groups, making the difference by age groups more ambiguous.

## Conclusions

Structural discrimination is the greatest barrier to receiving psychiatric help in Korea. Difference in the weight of barriers, however, is among age groups. As well as addressing structural issues for all, more tailored approaches may be required by generations to lower the gap. Further studies are needed to validate the factors associated with barriers to psychiatric service use.

## Data Availability

The corpus used for the analysis is not accessible.
